# Selection and validation of reference genes for normalizing qRT‒PCR gene expression studies in *Colletotrichum gloeosporioides* and interaction with the guava plants

**DOI:** 10.3389/fpls.2023.1235848

**Published:** 2023-11-23

**Authors:** Imran Ul Haq, Siddra Ijaz, Abeer Hashem, Graciela Dolores Avila-Quezada, Elsayed Fathi Abd_Allah

**Affiliations:** ^1^ Department of Plant Pathology, University of Agriculture Faisalabad, Faisalabad, Pakistan; ^2^ Centre of Agricultural Biochemistry and Biotechnology, University of Agriculture Faisalabad, Faisalabad, Pakistan; ^3^ Botany and Microbiology Department, College of Science, King Saud University, Riyadh, Saudi Arabia; ^4^ Facultad de Ciencias Agrotecnológicas, Universidad Autónoma de Chihuahua, Chihuahua, Chihuahua, Mexico; ^5^ Plant Production Department, College of Food and Agricultural Sciences, King Saud University, Riyadh, Saudi Arabia

**Keywords:** qRT−PCR, reference genes, *Colletotrichum gloeosporioides*, *Psidium guajava*, pathogenesis-related genes

## Abstract

Quantitative real-time PCR is used to quantify gene expression, even to detect low-level transcripts. It detects and quantifies the inoculum level of fungal pathogens in infected hosts. However, reliable expression profiling data require accurate transcript normalization against a stable reference gene. Hence, using stably expressed reference genes under variable conditions is paramount in gene expression analysis. In the current study, reference genes were selected and validated in *Colletotrichum gloeosporioides*, a guava canker and dieback pathogen. The reference gene selection and validation in *C. gloeosporioides* were evaluated for germinated conidia and mycelium (*in vitro*) and in infected guava (*Psidium guajava*) (interaction with host plant). The *CgCAL* gene was determined as a highly stable reference gene, followed by the *CgTUB2* in *C. gloeosporioides* for germinating conidia and mycelium. However, the *CgTUB2* gene was determined to be a highly stable reference gene, followed by the *CgCAL* for expression analysis during its interaction with the plant. Expression profiling revealed stable and constant relative expression patterns of selected reference genes for both PR genes by determining their relative transcript level. This study is the first to describe reference gene selection and validation to quantify target gene expression in *C. gloeosporioides*.

## Introduction

The genus Colletotrichum includes a broad array of economically important fungal pathogens that infect various host plant species (Yan et al., 2018; Iqra et al., 2022). Fungal biology, genomics, genetics, colonization, virulence factors, and interaction with host plants are prerequisites to mitigate compromised quality and consequent financial losses in the agricultural sector (Villa-Rivera et al., 2017; Babar et al., 2021). For this purpose, model species have been extensively studied for insight into infection stages and host interactions (Haq and Ijaz, 2020). Different techniques are involved in expression profiling, including northern blotting, semiquantitative real-time PCR, microarray, RNAseq, and quantitative real-time PCR (qRT−PCR) (Ijaz et al., 2020a). Among these, qRT-PCR is the most efficient technique for determining the relative levels of target gene expression in different samples (MaChado et al., 2015; Ijaz et al., 2019). A notable advantage of qRT-PCR over other conventional techniques is its ability to detect low starting material copies of the targeted gene’s mRNA (Huggett et al., 2005; Ijaz and Haq, 2020). Nonetheless, the results obtained through qRT-PCR depend upon the accuracy of target transcript normalization using appropriate reference genes for controlling significant experimental errors (Radonić et al., 2004).

Functional gene expression quantification of the targeted gene is crucial for comprehensive gene transcription and regulation studies through qRT-PCR (Marcial-Quino et al., 2016). A systemic study quantified crucial functional gene expression aspects using qRT-PCR for the targeted gene. This technique for detecting gene expression and profiling analysis is simple, fast, highly sensitive, and reproducible (Hao et al., 2014; Haq et al., 2019). However, data analysis requires suitable reference genes, pivotal in generating reliable qRT-PCR results. In contrast, incompetent reference genes produce misleading expression data (Galli et al., 2015). A reliable reference gene should be constitutively expressed in experimental samples, with unaltered expression under diverse experimental sets (Galli et al., 2015). For Colletotrichum members, several commonly used reference genes have been documented; for example, in *Colletotrichum higginasianum*, the actin (*ACT*) gene was used to quantify and normalize fungal growth and transporter (*MFS*) gene expression (Liu et al., 2017). However, in *Colletotrichum fructicola*, the Ras-related protein *gene* (*CfRrp*) showed better stability (Chen et al., 2022) in quantifying target gene expression. However, to our knowledge, no literature and studies have been found for the reference gene selection to normalize qRT-PCR gene expression analysis in *Colletotrichum gloeosporioides.*

Studies have illustrated that reference gene expression stability varies considerably between species and could differ across tissue types, stages of development, and experimental conditions (Guenin et al., 2009). Therefore, a constitutively expressed gene is essential for obtaining reliable quantitative real-time PCR results, which should be stable at all growth stages and may serve as internal controls. The most suitable and preferred technique for analyzing gene expression is qRT-PCR. However, in *C. gloeosporioides*, no suitable, reliable reference genes have been reported for qRT-PCR, but their presence is expected in different experimental conditions, which need to be identified. *C. gloeosporioides* is a predominant fungal pathogen of guava plants (Lu et al., 2018; Iqra et al., 2022). It is responsible for leaf and other severe guava plant diseases, notably leaf blight, brown blight, and anthracnose (Wang et al., 2016; Haq and Ijaz, 2019; Haq et al., 2021). It is the first study to select and validate suitable reference genes in C. gloeosporioides for transcript normalization in qRT-PCR. Housekeeping genes, including actin (*ACT*), Glyceraldehyde-3-phosphate dehydrogenase (*GAPDH*), 18S ribosomal RNA *(18S rRNA)*, calmodulin (*CAL*), and β-tubulin (*TUB2*), are considered the most appropriate candidate reference genes for fungal plant pathogens because of their involvement in essential cellular functions (Galli et al., 2015; Haq and Ijaz, 2020). In the present study, four candidate reference genes were appraised for their identification and suitability for transcript normalization in *C. gloeosporioides* at germinating conidia and mycelial stage and interaction with *Psidium guajava*. Their stability evaluation was performed with Excel-based software. These data analyses extensively validated the putative reference gene, which can help further gene expression studies associated with this fungus.

## Materials and methods

### Fungal culture

Germinating conidia and mycelial stage of fungal pathogen *Colletotrichum gloeosporioides* were considered for selecting suitable reference genes for transcript normalization in qRT−PCR. *C. gloeosporioides* was cultured with a sterile needle on a Potato Dextrose Agar (PDA) medium to get germinate conidia and mycelium. For this purpose, single spore and hyphae tip methods were employed. The culture plates were incubated at 27°C under a dark regime for 48-72 hrs, and growth was observed. Microscopical features were examined under stereomicroscopy.

### Plant inoculation

For reference gene selection, while the interaction of *C. gloeosporioides* with guava pants, fungal inoculation of plants was performed. A Guava cultivar named “Large Sorahie” (Pyriform) was used in this experiment. Plants were raised in the research area of the Department of Plant Pathology, University of Agriculture Faisalabad, under the greenhouse-controlled condition. Guava plants at the seedling stage (six months old) were inoculated by spraying the plants with a spore suspension (150 ml), which was adjusted to a concentration of 1×10^6^ spores ml^−1^ (Chen et al., 2021).

### RNA isolation

Total RNA was isolated from germinated conidia and mycelium of *C. gloeosporioides* and inoculated guava leaves sampled at two, three, and four weeks postinoculation. The GeneJET Plant RNA Purification kit (Thermo Scientific USA) was used for RNA extraction. The quantification of extracted RNA was determined using a UV-visible NANODROP 8000 Spectrophotometer (Thermo Scientific USA); however, integrity was analyzed by gel electrophoresis using 1.5% agarose. The isolated RNA from each sample was treated with a RapidOut DNA Removal kit (Thermo Scientific USA) to remove genomic DNA (gDNA).

### cDNA synthesis

Treated RNA samples were reverse transcribed with RevertAid M-MuLV RT using a RevertAid First Strand cDNA Synthesis Kit (Thermo Scientific USA) in a final volume, as per the manufacturer’s recommendations, 20-fold diluted and quantified by UV visible NANODROP 8000 Spectrophotometer (Thermo Scientific USA).

### Quantitative real-time PCR analysis

Five housekeeping genes were assessed as candidate reference genes in *C. gloeosporioides*, including calmodulin (*CgCAL*), Glyceraldehyde-3-phosphate dehydrogenase (*CgGAPDH*), 18S rRNA, β-tubulin (*CgTUB2*), and actin (*CgACT*). The primers employed for qRT-PCR analysis were designed using PrimerQuest. To verify their specificity for *C. gloeosporioides*, we used general PCR to test amplification in guava. Only primers with no amplification for guava were retained for qRT−PCR analysis (**Table 1**). This analysis was carried out in a CFX96 Touch Real-Time PCR detection system using a 96 × 0.2 ml plate (Bioplastics Netherlands) with a 5 μl total reaction volume for each sample, containing cDNA (1 μl), Maxima Syber green/ROX/qPCR master mix (2.5 μl) (Thermo Scientific USA), and 1 μM of each primer. For the negative control, water was used in the reaction instead of cDNA (no-template control). The thermal profile for qRT−PCR was denaturation at 95°C (60 sec), 40 amplification cycles at 95°C (20 sec), and an annealing/extension step at 60°C (30 sec). The melting curve analysis was employed at 60°C to 95°C. For each sample, three biological replicates and three technical replicates for each well were made. No-template controls (NTCs) (cDNA zero) were also assessed for each primer pair of candidate reference genes. The data were analyzed using CFX Manager ™ Software v. 3.1 (Bio-Rad Laboratories, Inc. USA).

**Table 1:** The primers used in the study.

Gene name/Symbol	primers (5′ to 3′)	Accession No.	Amplicon size (bp)	Source
***β-Tubulin (tub2)***	**F** AGATTGGTGCTGCCTTCTG **R** CTTCGTTGAAGTAGACGCTCAT	MN339477	117	Present study
***Glyceraldehyde 3-phosphate dehydrogenase (gapdh)***	**F** GGTGCCAAGAAGGTCATCAT **R** GTTGTGCAAGAAGCGTTGG	MT573960	119	Present study
***Actin (act)***	**F** ATGTGCAAGGCCGGTTT **R** CTTCTGGCCCATACCAATCAT	JX009502	105	Present study
***Calmodulin (cal)***	**F** CGATGGCCAAATCACTACAAAG **R** CATAGTCAGGAACTCGGGAAAG	MN308244	145	Present study
***Diacylglycerol acyltransferase (DGAT1)***	**F** GTGAGAGTCTGGGTGCTTACTG **R** CGATCAAGGGAGAGTAGACGTG			Liang et al. (2018)
***3-Dehydroshikimate dehydratase (qutC)***	**F** ATGCCTTCACGCCTGGGTAT **R** TTTGCTGCCATGTCCATCTTGT			Liang et al. (2018)

### Stability analysis and validation of selected reference genes

Three mathematical algorithms, geNorm (https://genorm.cmgg.be), NormFinder (https://moma.dk/normfnder-sofware), and BestKeeper (https://www.gene-quantifcation.de/bestkeeper.html), were applied to assess the candidate reference genes’ expression stability for transcript normalization. The best-ranked reference genes in *C. gloeosporioides* were assessed; the Cq (quantification cycle) conversion value to relative quantitates, except for the BestKeeper algorithm, was made. The overall comprehensive ranking was generated through the RefFinder tool (https://heartcure.com.au). The selected reference genes were validated for normalizing the expression data of qRT−PCR in *C. gloeosporioides* using the diacylglycerol acyltransferase (*DGAT1*) gene and 3-dehydroshikimate dehydratase (*qutC*) gene in qRT−PCR analysis. These pathogenicity-related genes were documented by Liang et al. (2018) in a fungal species of the *C. gloeosporioides* species complex.

## Results

### Expression analysis

**Figure 1:**
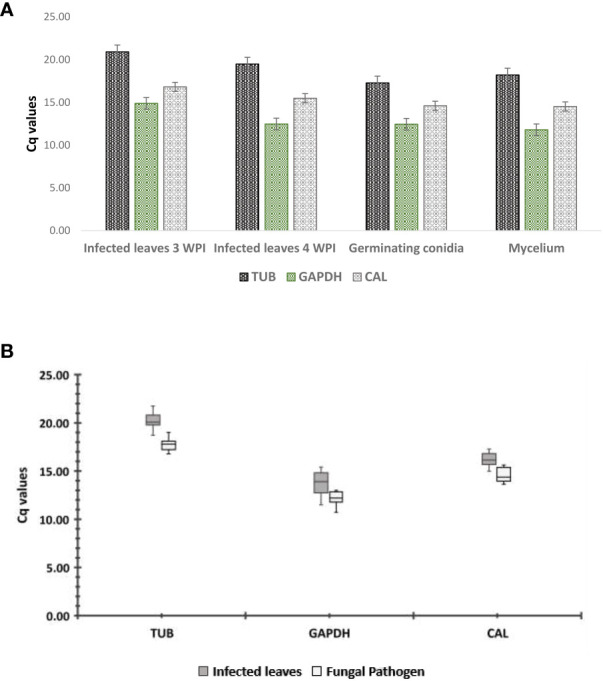
qRT−PCR analysis: **(A)** expression profiling of candidate reference genes in *Colletotrichum gloeosporioides* using qRT−PCR, **(B)** expression analysis of candidate genes in pathogen and infected leaves. Boxes denote lower and upper quartiles of the cycle threshold range with medians indicated; whisker caps symbolize minimum and maximum values. White boxes correspond to pathogen samples and gray boxes to infected leaf samples.

Target DNA fragments were amplified from the cDNA of *C. gloeosporioides* by tested primers. Among these, 18S rRNA gave amplification in noninfected guava plants; however, the remaining four tested primers showed no amplification in noninfected guava leaves, which confirmed the absence of *C. gloeosporioides* in the control. Hence, the 18S rRNA gene was not used as a candidate reference gene for the qRT−PCR study in *C. gloeosporioides*. Melting curve analysis gave a single peak in all test primers and validated the primer specificity for qRT-PCR analysis. The Cq value was used to determine the gene expression levels, and it has an inverse relationship with gene expression, as a high Cq value reflects low gene expression and vice versa. The gene expression directly in the RNA sample of a fungal pathogen and RNA samples in an infected plant (related to the concentration of that fungal pathogen) cannot be exactly matched. However, it could be similar in these samples. That is why the expression analysis directly in the pathogen and the pathogen in the infected plant could not be compared (Song et al., 2019). The expression levels of the candidate reference genes, *CgCAL*, *CgTUB2*, and *CgGAPDH*, were similar in all samples (**Figure 1**), except for *CgACT*, which showed upregulation in infected plant material. Therefore, we discarded this housekeeping gene in further analyses of selecting reference gene(s) for transcript normalization in *C. gloeosporioides*. For the statistical evaluation of the candidate reference genes, three statistical algorithms were employed to rank the *CgCAL*, *CgTUB2*, and *CgGAPDH* genes based on expression stability. For expression stability analysis, we selected infected leaves of the guava plant after the third and fourth weeks postinoculation. The pathogen’s inoculum level in infected leaves of these periods was higher than that of infected leaves two weeks postinoculation (WPI) based on symptoms that appeared.

### Stability analysis of candidate reference genes for normalizing qRT−PCR in *Colletotrichum gloeosporioides*

A study was carried out to select reference genes (*CgGAPDH*, *CgCAL*, and *CgTUB2*) for normalizing qRT−PCR expression analysis of *C. gloeosporioides* at four different levels (germinated conidia, mycelium, and infected leaves of guava plants at three and four weeks after inoculation). Three Excel-based statistical software programs were used to select a highly stable reference gene for normalization in expression profiling. The generated Cq values were converted into relative quantities for the analysis. The geNorm analysis revealed that the candidate reference genes *CgTUB2* and *CgCAL* in combination were projected to have the least stability values, “M,” in germinating conidia and mycelium (0.040) and infected leaves (0.025). They ranked as the best reference genes with the highest stability for accurate data normalization in the qRT-PCR analysis of *C. gloeosporioides* (**Table 2**).

**Table 2:** geNorm analysis expression stability of candidate reference genes (a) in germinated conidia and mycelium of *Colletotrichum gloeosporioides* (b) in infected leaves of guava plants.

(a)			(b)		
Ranking order	Candidate Reference Genes	Average expression Stability value " M"	Ranking order	Candidate Reference Genes	Average expression Stability value " M"
1	*CgTUB2 | CgCAL*	0.040	1	*CgTUB2 | CgCAL*	0.025
2	*CgGAPDH*	0.212	2	*CgGAPDH*	0.244

The descriptive data of the candidate reference genes based on expression in different samples are given in **Table 3**. In germinated conidia and mycelium of *C. gloeosporioides*, the candidate reference gene *CgTUB2* with the lowest CV (% CP) ± SD value of 3.43 ± 0.61 was identified as the most stable reference gene for transcript normalization. However, *CgGAPDH* (5.52 ± 0.67) and *CgCAL* (4.83 ± 0.71) were ranked as the second and third reference genes, respectively, with stable expression for normalizing qRT−PCR analysis. In the case of infected leaves of guava plants, the ranking was observed; *CgCAL* ranked as the top best reference gene, with a CV (% CP) ± SD score of 3.86 ± 0.66, followed by *CgTUB2*, with a value of 4.09 ± 0.78. However, in the BestKeeper ranking, *CgGAPDH* was proven to be a reference gene with unstable expression by scoring an 8.82 ± 1.21 value because a gene with SD >1 is considered an unstable expression (**Table 3**).

**Table 3:** BestKeeper analysis of the expression stability of candidate reference genes: (a) in germinated conidia and mycelium of *Colletotrichum gloeosporioides* and (b) in infected leaves of guava plants.

(a)				(b)			
	Candidate Reference Genes				Candidate Reference Genes		
	*CgTUB2*	*CgGAPDH*	*CgCAL*		*CgCAL*	*CgTUB2*	*CgGAPDH*
geo* Mean [CP]	17.75	12.11	14.56	geo Mean [CP]	16.16	20.20	13.63
AR* Mean [CP]	17.77	12.14	14.58	AR Mean [CP]	16.18	20.22	13.70
min [CP*]	16.79	10.73	13.62	min [CP]	14.98	18.70	11.49
max [CP]	19.00	13.00	15.62	max [CP]	17.26	21.74	15.41
std dev [+/- CP]	0.61	0.67	0.71	std dev [+/- CP]	0.66	0.78	1.21
CV* [% CP]	3.43	5.52	4.83	CV [% CP]	3.86	4.09	8.82
Coeff. of corr.[r]	0.411	0.487	0.863	Coeff. of corr.[r]	0.850	0.946	0.976
Coeff. of det.[r2]	0.168	0.237	0.744	Coeff. of det.[r2]	0.722	0.89	0.952

*geo Mean, Geometric Mean; AR mean, Arithmetic mean; CV, Coefficient of Variance; CP, Crossing Point.

NormFinder analysis showed that the *CgCAL* gene had the most stable expression, with a stability value of 0.020 in germinating conidia and mycelium; this gene’s same stability pattern was observed in infected leaves, with a stability value of 0.013. The ranking of candidate reference genes based on expression stability in germinating conidia and mycelium was *CgCAL*> *CgTUB2*>*CgGAPDH*. The same ranking was found in the NormFinder analysis of infected leaves of the guava plant (**Table 4**).

**Table 4:** NormFinder analysis of the expression stability of candidate reference genes: (a) in germinating conidia and mycelium of *Colletotrichum gloeosporioides* and (b) in infected leaves of guava plants.

(a)			(b)		
Ranking order	Candidate Reference Genes	Stability value	Ranking order	Candidate Reference Genes	Stability value
1	*CgCAL*	0.020	1	*CgCAL*	0.013
2	*CgTUB2*	0.045	2	*CgTUB2*	0.057
3	*CgGAPDH*	0.296	3	*CgGAPDH*	0.353

In germinating conidia and mycelium, the results obtained using BestKeeper indicated that the reference gene *CgTUB2* was stable in *C. gloeosporioides*. The result from NormFinder revealed the *CgCAL* gene as a stable reference gene, although geNorm gave the combination of reference genes, *CgTUB2* and *CgCAL*, for normalization of gene expression analysis. However, the comprehensive ranking of candidate reference genes was determined using the RefFinder program, which ranked the *CgCAL* gene as the most stable reference gene for expression analysis in *C. gloeosporioides* in all experimental sets (germinating conidia, mycelium, and infected leaves of guava plants after three weeks and four weeks of inoculation). The comprehensive ranking order, *CgCAL*>*CgTUB2*>*CgGAPDH*, for stable reference genes for transcript normalization of qRT−PCR in *C. gloeosporioides*, was found to be matched with the ranking order given by the NormFinder method (**Table 5**). Hence, all statistical programs’ overall results determined the *CgCAL* gene, a highly stable reference gene, followed by the *CgTUB2* reference gene, for transcript normalization in the qRT−PCR analysis in *C. gloeosporioides* for germinating conidia and mycelial stage. However, the *CgTUB2* gene was determined to be a highly stable reference, followed by the *CgCAL* reference gene for expression analysis during its interaction with the plant.

**Table 5:** Expression stability ranking of candidate reference genes expressed (a) in germinating conidia and mycelium of *Colletotrichum gloeosporioides* and (b) in infected leaves.

(a)				(b)			
Statistical method	Ranking order			Statistical method	Ranking order		
	1	2	3		1	2	3
BestKeeper	*CgTUB2*	*CgGAPDH*	*CgCAL*	BestKeeper	*CgCAL*	*CgTUB2*	*CgGAPDH*
NormFinder	*CgCAL*	*CgTUB2*	*CgGAPDH*	NormFinder	*CgCAL*	*CgTUB2*	*CgGAPDH*
geNorm	*CgTUB2 | CgCAL*	*CgGAPDH*		geNorm	*CgTUB2 | CgCAL*	*CgGAPDH*	
Comprehensive Ranking	*CgCAL*	*CgTUB2*	*CgGAPDH*	Comprehensive Ranking	*CgTUB2*	*CgGAPDH*	*CgCAL*

### Expression profiling of pathogenicity-related genes for *Colletotrichum gloeosporioides* for validating selected reference genes

**Figure 2:**
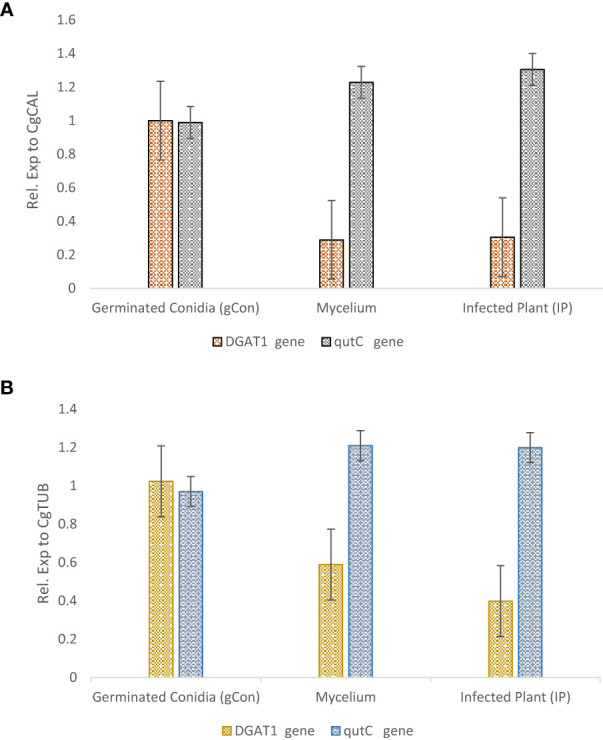
Expression profiling of *DGAT1* and *qutC* target genes in *Colletotrichum gloeosporioides* for germinating conidia, mycelium, and interaction with guava plants. The selected reference genes (*CgCAL* and *CgTUB2*) were used for data normalization: **(A)** *CgCAL*, **(B)** *CgTUB2*.

For selected reference gene validation for normalizing the gene expression data in *C. gloeosporioides*, two pathogenesis-related genes, Diacylglycerol acyltransferase (*DGAT1*) and *qutC*, were selected. The expression profiling of both genes was performed using qRT-PCR analysis by adopting the earlier profiles. The expression of these genes was determined relative to selected reference genes (*CgCAL* and *CgTUB2*) for normalization. *DGAT1* gene expression was higher in germinating conidia, but its conspicuous downregulation was observed in mycelium. However, *qutC* gene expression was higher in the infected plant sample (4^th^-week postinoculation), followed by mycelium, which supports the role of this gene in infection (**Figure 2**). Expression profiling of PR genes for validating the selected reference genes for accurate transcript normalization by determining the relative transcript level of PR genes revealed stable and constant relative expression pattern of selected reference genes for both PR genes.

## Discussion

Quantitative real-time PCR has developed an imperative technique for studying transcript profiling (Wang et al., 2017). However, the reliability of the results depends upon the reliable reference genes vis-à-vis their expression stability for normalizing expression data (Huang et al., 2018) that should not be altered under varied experimental conditions (Melgar-Rojas et al., 2015; Ijaz et al., 2020b). Under varying experimental conditions, it is ambiguous to use traditional reference genes for normalizing target gene expression without prior investigation of their stability (Galli et al., 2015; Nasir et al., 2023).

There is no report on selecting and validating reliable reference genes for qRT−PCR analysis of target gene expression in *C. gloeosporioides* and during its interaction with host plants. The specificity of endogenous reference genes is a prerequisite for studying plant-pathogen interactions. Hence, in addition to analyzing candidate reference gene primer specificity for *C. gloeosporioides* cDNA amplification, the cDNA of the noninfected guava plant was also subjected to PCR analysis using these primers. The 18S rRNA gene was eliminated for qRT−PCR to study *C. gloeosporioides* gene expression and validate the best reference gene for gene expression analysis on two critical developmental stages, germinating conidia and mycelium, and during its interaction with the guava plant because it gave amplification in the noninfected leaves of guava plants. Statistical software for determining reliable reference genes in fungi and experimental samples influences the results (Song et al., 2019). Therefore, we chose *C. gloeosporioides* samples and leaf-inoculated guava plants at different stages to detect and validate the reference gene for qRT−PCR.

For qRT-PCR analysis, the candidate genes were evaluated by Cq values. We analyzed the expression stability of each candidate reference gene in germinating conidia and mycelium and three- and four-week-infected guava leaves. The candidate reference gene *CgACT* was unsuitable for gene expression stability analyses because it showed a higher expression level in infected leaves than in germinating conidia and mycelium samples. In infected plants, fungal pathogen inoculation varies considerably during infection (Vieira et al., 2016). The gene expression of *C. gloeosporioides* during the germinating conidia and mycelium and in postinoculated leaves of guava plants showed increased expression levels of the *CgTUB2* gene.

Several statistical approaches select reference genes with stable expression across biological samples. We used geNorm, NormFinder, and BestKeeper for stability analysis. However, all these algorithms gave varied results, so the RefFinder method was used for comprehensive analysis to avoid the contradiction of individual methods. Based on comprehensive ranking using Ref Finder, *CgCAL* was selected as the top-ranked, most reliable candidate reference gene for normalizing qRT−PCR of target gene expression in *C. gloeosporioides*, followed by the *CgTUB2* reference gene. However, *CgTUB2* was selected as the top-ranked, most reliable candidate reference gene for qRT−PCR of *C. gloeosporioides*’ target gene expression normalization in infected leaves, followed by the *CgCAL* reference gene. Our results supported the findings of Vieira et al. (2016). They validated the β-tubulin gene as a stable reference gene for expression studies in *C. kahawae*. However, Paolinelli-Alfonso et al. (2016) recommended β-tubulin, the best reference gene for normalization in *Lasiodiplodia theobromae*. Therefore, this study is the first to evaluate the selection and validation of reliable reference genes for normalization in *C. gloeosporioides* expression analysis.

## Data availability statement

The datasets presented in this study can be found in online repositories. The names of the repository/repositories and accession number(s) can be found in the article/supplementary material.

## Author contributions

Conceptualization, IH and SI. Data curation, IH and SI. Formal analysis, IH and SI. Funding acquisition, EA_A. Writing – original draft, IH and SI. Writing – review & editing, IH, SI, EA_A, AH, and GA-Q. All authors contributed to the article and approved the submitted version.
